# A data-driven financial justification for an outpatient infectious diseases pharmacist

**DOI:** 10.1017/ash.2025.10051

**Published:** 2025-07-11

**Authors:** Justin J. Kim, Laura N. Hernandez Guarin, Jonathan Contreras, Mary K. Hayden, Sarah Y. Won

**Affiliations:** 1 Division of Infectious Diseases, Department of Internal Medicine, RUSH University Medical Center, Chicago, IL, USA; 2 Division of Clinical Microbiology, Department of Pathology, RUSH University Medical Center, Chicago, IL, USA

## Abstract

We present a financial justification for an outpatient infectious diseases pharmacist, based on cost savings from decreases in length of stay for patients with *Staphylococcus aureus* infections and additional revenue generated by physicians and pharmacists while following patients discharged on outpatient parenteral antimicrobial therapy.

## Introduction

Outpatient infectious diseases (ID) pharmacists are increasingly required to ensure the delivery of high quality care to patients with infections in the ambulatory setting.^1,2^ They can perform medication adjustments and changes, monitor laboratory test results to improve patient safety, coordinate infusions, and submit insurance approvals or appeals,^3^ and their involvement can result in cost savings.^4,5^ However, the financial justification of these roles can be challenging.^6^ Here we provide a data-driven financial justification of an outpatient ID pharmacist, which was the basis for approval of the position at our institution.

## Methods

We retrospectively examined a 50% random sample of 1146 unique inpatients seen by the general ID consult team (ie, CPT codes 99254, 99255, 99222, 99223, 99232, and 99233) from April to September 2023 at our 671-bed urban medical center. Based on the most recent ID note, we manually collected the final ID diagnosis resulting in the longest antibiotic duration (ie, only one ID diagnosis per patient), antibiotic end date, date of discharge, and noted if the patient was enrolled in our outpatient parenteral antimicrobial therapy (OPAT) program.

To identify potential savings for our medical center, we examined the following patient populations whose length of stay could potentially be decreased with support from an outpatient ID pharmacist.

1. Patients admitted for *Staphylococcus aureus* bacteremia without a safe discharge plan (e.g., not an OPAT candidate because of active injection drug use or unstable housing): To estimate the maximum decrease in length of stay, we subtracted the date on which patients were medically ready for discharge from the actual date of discharge. We suggested that an outpatient ID pharmacist could facilitate an earlier discharge using various strategies (e.g., coordinating dalbavancin in the outpatient setting, prescribing linezolid with appropriate monitoring), rather than requiring admission for their entire 4–6 week course of intravenous antibiotics.
2. Patients admitted for methicillin-resistant *S. aureus* (MRSA) bacteremia, prosthetic joint infection, or spine infection awaiting optimization of vancomycin dosing: To estimate the maximum decrease in length of stay, we counted the number of patients discharged on vancomycin, who had a plasma trough that was out of range (eg, 15–20 mcg/ml) anytime during their admission, assuming that 2 days would be needed to optimize dosing following a subtherapeutic or supratherapeutic trough. We suggested that an outpatient ID pharmacist could facilitate an earlier discharge by optimizing vancomycin dosing in the outpatient setting, rather than optimizing vancomycin dosing in hospital as we currently do.

We used the variable direct cost per day of the medical-surgical units of our institution to estimate the savings from these reductions in length of stay.^7^ In addition to savings, we identified opportunities for revenue for our medical center from the ambulatory setting.

1. Physician visits for patients discharged with >14 days of antibiotics without ID follow-up: We counted the number of patients discharged with >14 days of antibiotics (i.e., oral or intravenous) without ID follow-up. We reasoned that at least one follow-up visit would be justifiable for these patients, with 50% billed for CPT code 99214, and 50% billed for CPT code 99215). We also reasoned that an outpatient ID pharmacist could help increase the show rate to these follow-up visits through telehealth.
2. Outpatient ID pharmacist telehealth for OPAT patients discharged with >14 days of antibiotics: We counted the number of OPAT patients discharged with >14 days of antibiotics and the total of antibiotic weeks. We assumed one telehealth visit per antibiotic week, subtracting one week per patient to account for follow-up with a physician. For patients on OPAT, a weekly call to discuss lab results and to monitor for antibiotic side effects would be a way of providing aspirational care and may be a more appropriate task for an outpatient ID pharmacist than a nurse or a physician.

We calculated the net income by subtracting the salary of outpatient ID pharmacist from the savings and revenue.

## Results

We examined 573 unique patients evaluated by 16 ID providers between April and September 2023, 18% of whom were discharged on OPAT. The most common ID diagnoses included orthopedic (23%), gastrointestinal (13%), skin and soft tissue (12%), respiratory tract (6%), urinary tract (6%), systemic viral (6%), central nervous system (4%), and cardiovascular (2%) infections, as well as *S. aureus* (4%) and other (4%) bacteremias (eg, catheter-related bloodstream infections). Twelve percent had no infection, 3% died or were discharged to hospice, and 3% did not have a final diagnosis by the end of September 2023.

Of the 31 patients with a diagnosis of *S. aureus* bacteremia, prosthetic joint infection, or spine infection, 3 were persons who inject drugs, 1 of whom was admitted for an additional 40 days over 6 months due to the lack of a safe discharge plan (ie, not a candidate for OPAT because of active injection drug use), despite being medically ready for discharge sooner. Of the other 28 patients with *S. aureus* infection, 11 were admitted for MRSA, 4 of whom were discharged on vancomycin; all of these had a trough that was out of range during their admission. There were 160 patients discharged with >14 days of antibiotics (ie, oral or intravenous) remaining, 58 of whom had no ID follow up. There were 74 patients on OPAT discharged with >14 days of antibiotics remaining, amounting to a total of 358 antibiotic weeks. Assuming one physician visit per antibiotic course would replace the pharmacist encounter for that week left 284 (ie, 358 minus 74) weeks during which a telehealth visit from the outpatient ID pharmacist would be appropriate. We assumed that these data from a 50% sample of 6 months of consults could be multiplied by four to estimate the annual savings and revenue associated with an outpatient ID pharmacist. These savings and revenue more than covered the salary of an outpatient ID pharmacist with a net income of +$27,901. These results are summarized in Table 1.

**Table 1.** Financial summary for outpatient ID pharmacist justification

*Savings*			
	Number of patients	Decrease in LOS per patient (days)	Total savings
Patients admitted with *Staphylococcus aureus* infection without a safe discharge plan	4	40	160×a
Patients admitted with MRSA infection awaiting optimization of vancomycin dosing	16	2	32×a
*Revenue*			
	Number of encounters	Charge per encounter	Total revenue in USD
Physician visits for patients discharged with >14 days of antibiotics without ID follow-up	228	99214: b 99215: c	116×b + 116×c
Outpatient ID pharmacist telehealth encounters for OPAT patients discharged with >14 days of antibiotics	1136	99211: d	1138×d
*Salary*			
Outpatient ID pharmacist salary			e
**Net income**			**192×a + 116×b + 116×c + 1138×d—e = +$27,901**

ID = infectious diseases, LOS = length of stay, MRSA = methicillin-resistant Staphylococcus aureus; *a* = variable direct cost per day for medical-surgical unit, *b* = average charge for CPT code 99214 minus deduction, *c* = average charge for CPT code 99215 minus deduction, *d* = average charge for CPT code 99211 minus deduction, *e* = outpatient ID pharmacist salary with fringe, USD = US dollars.

## Discussion

We have outlined a feasible and convincing approach for justifying an outpatient ID pharmacist that could be adopted across institutions with a relatively minimal burden of data collection. Our justification hinges on well-established metrics including length of stay and clinical revenue. Strengths of our approach included the manual collection of accurate patient-specific data from our medical center, which more persuasively conveyed to our leadership the need for this role than the experience of other institutions, and that this analysis can be reproduced at most institutions. Moreover, similar data could be used to monitor prospectively the output of the pharmacist once hired, by which these data could be fine-tuned in the future. Potential weaknesses included the reliance of this proposal on savings as opposed to revenue and the assumption that this random sample was representative of the entire year. Without the ability to compare lengths of stay with and without the outpatient ID pharmacist, it is impossible to conclude a causal relationship between the pharmacist and decreased lengths of stay, though this could be a future study to assess the effectiveness of the role (eg, using a time-interrupted series). To our knowledge, this is the first report proposing a financial justification for an outpatient ID pharmacist, which—above and beyond the business case—aligns with the standard of care for patients with infections in the ambulatory setting.
